# T.H.R.O.B.V.S. Score – A Comprehensive Model to Predict the Surgical Complexity of Renal Cell Carcinoma With Tumor Thrombus

**DOI:** 10.3389/fonc.2022.900550

**Published:** 2022-06-23

**Authors:** Kai Wang, Zhuo Liu, Peng Hong, Yan-chun Qin, Xun Zhao, Hong-xian Zhang, Cheng Liu, Li-yuan Ge, Lu-lin Ma

**Affiliations:** Department of Urology, Peking University Third Hospital, Beijing, China

**Keywords:** nephrectomy, thrombectomy, prediction model, surgical complexity, renal cell carcinoma

## Abstract

**Background:**

To propose a quantitative model for predicting the surgical complexity of patients with renal cell carcinoma (RCC) and venous tumor thrombus (VTT).

**Method:**

The clinical data of 226 cases of RCC with VTT in Peking University Third Hospital from January 2014 to August 2020 were retrospectively analyzed. Seven indicators were selected to establish the T.H.R.O.B.V.S. system, including alkaline phosphatase, tumor thrombus height, maximum tumor diameter, obesity, bland thrombus, vascular wall invasion, and side. Each indicator was assigned with 0, (1), and 2 points, and the total scores of 0~2, 3~5, and ≥6 were set as the low-, middle-, and high-risk groups, respectively. The surgical complexity was compared and validated among groups.

**Results:**

As the risk increased, the proportion of open surgery significantly increased (P<0.001). The operation time (P<0.001), intraoperative blood loss (P<0.001), blood or plasma transfusion (P<0.001), and hospitalization (P<0.001) increased significantly. The postoperative complications (P<0.001), including notable complications (≥Clavein-Dindo II, P<0.001), were significantly different, and similar trends were shown in the validation group.

**Conclusion:**

The T.H.R.O.B.V.S. scoring system is a quantifiable and satisfactory model to predict the surgical complexity and perioperative management of RCC with VTT.

## Introduction

Renal cell carcinoma (RCC) is a malignant tumor originating from the renal tubule epithelial system, accounting for 2%~3% of adult tumors (1). In the last two decades, the prevalence of RCC has increased by 2% worldwide, especially in developed countries, becoming the ninth most common tumor in the United States (1, 2). Some studies have shown genetic factors, unhealthy lifestyle (tobacco smoking, alcohol consumption, physical inactivity), obesity, hypertension, diabetes, chronic kidney disease, environmental factors, and chemical exposure (trichloroethylene, aristolochic acid, etc.) are associated with RCC (3). Advances in screening programs and techniques allow more patients to be detected and treated in the early stage, and locally advanced or metastatic cases are no longer considered noncurable. As a result, the overall 5-year survival rate for RCC has increased from 46.8% in 1977 to 76% in 2020 in the USA (3, 4).

Notably, about 4%~10% of RCC cases have venous tumor thrombus (VTT) during progression (1, 5), which indicates a poor prognosis with a 5-year survival rate of 29% if untreated. Radical nephrectomy (RN) and tumor thrombectomy are the core curing methods and have significantly improved the 5-year survival rate of patients to 40%~60% (6, 7). However, RN and thrombectomy are still some of the most challenging urological operations. The surgical complexity and trauma vary based on the primary tumor and tumor thrombus characteristics (5). For instance, surgeons tend to remove the affected kidney and tumor thrombus directly under partial blockage of blood flow for cases with well-defined primary tumors and short tumor thrombi without vascular wall invasion. Foley catheters may be used to pull out the tumor thrombus (8). In some advanced Mayo IV cases, cardiopulmonary bypass (CPB) and open surgery may be chosen, which means a longer operation time, more intraoperative bleeding, extended hospital stays, a higher possibility of postoperative complications, and poor prognosis, which poses a significant challenge to both surgeons and patients (9).

Mayo grading is widely accepted for classifying tumor thrombi in RCC (5). In Grade 0, the tumor thrombus is limited in the renal vein; Grade I, the tumor thrombus extends into the inferior vena cava (IVC), and the tip is less than 2 cm from the opening of the renal vein; Grade II, the distance between the tip and the opening of the renal vein is more than 2 cm but lower than the level of the hepatic vein; Grade III, the tumor thrombus extends to the level of intrahepatic IVC but is lower than the diaphragm; and Grade IV, the tumor thrombus extends above the level of the diaphragm.

Predicting the complexity of operations conveniently and accurately before surgery has always been of great significance in clinical research. However, there is no quantitative tool to help surgeons make a proper determination with patients in advanced RCC with VTT (10). Most surgical decisions currently rely on qualitative indicators (11–13), which are far from standardized. It is detrimental to the reproducibility of treatment decisions and the growth of less-experienced surgeons. In this study, we tried to develop a surgical complexity prediction system for RCC with VTT patients based on some clinical and imaging data, named T.H.R.O.B.V.S. score, to predict the risk stratification of surgical complexity (including operation time, blood loss, hospitalization time, and postoperative complications) quantitively before the operation, combining with our considerable experience and previous literature reports. It may help urologists make proper perioperative preparations (preoperative blood preparation, critical steps processing, ICU monitoring, etc.), facilitate reproducible decision making, and good doctor-patient communication.

## Material and Method

### Ethics Approval

This study complied with the Declaration of Helsinki and was approved by the Ethics Committee of our institute (Approval No.: IRB00006761-M2018178).

### Patient Selection

**Figure 1:**
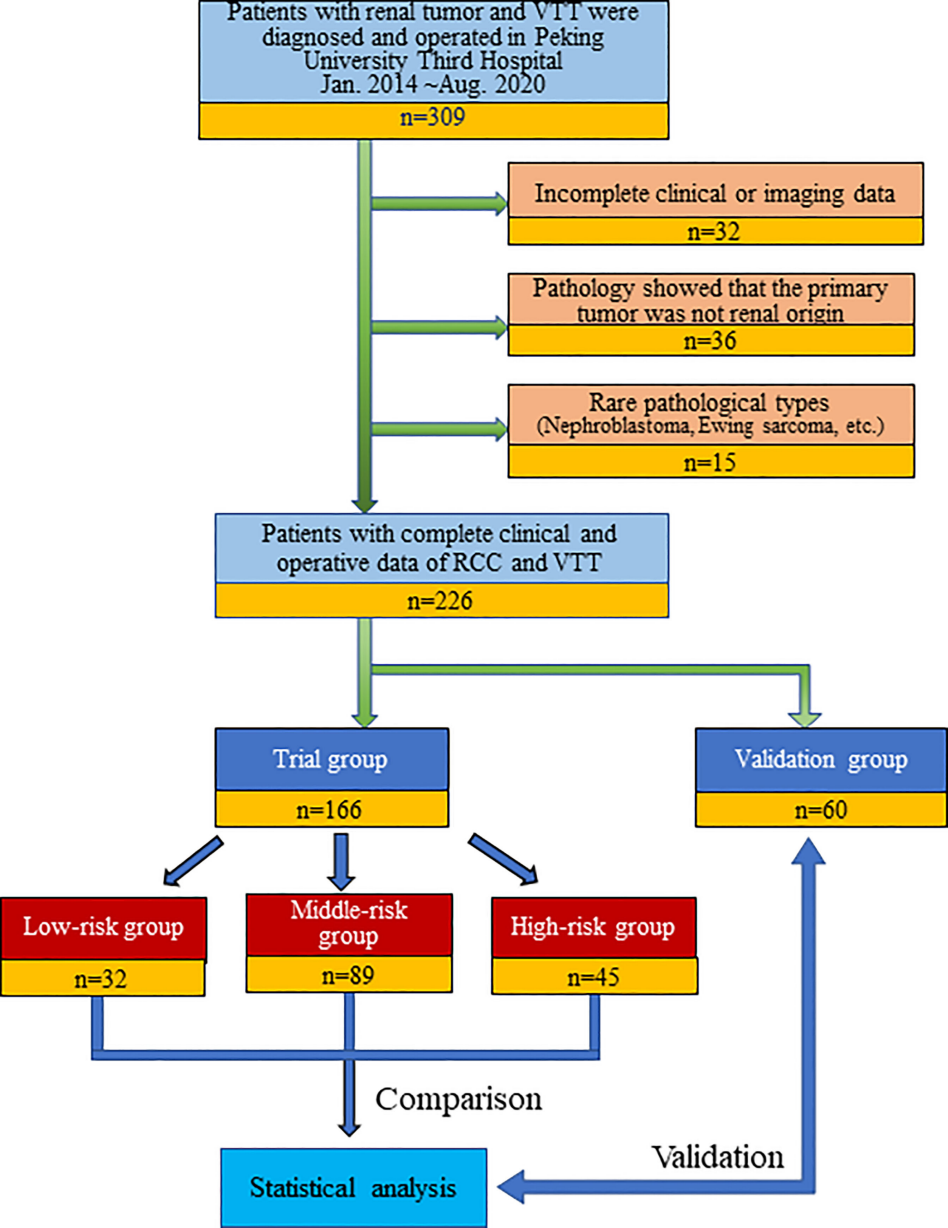
Flow chart of study cohort selection and research strategy.

We retrospectively continuously collected the clinical and imaging data of all patients (309 cases) diagnosed with RCC combined with renal vein tumor thrombus (RVTT) or inferior vena cava tumor thrombus (IVCTT) who underwent surgery at Peking University Third Hospital, a large Chinese RCCVTT medical center, from January 2014 to August 2020. Patients with incomplete clinical or imaging information, recurrent tumor thrombectomy, and postoperative pathology confirming that the primary tumor was non-RCC (urothelial carcinoma, leiomyosarcoma, adrenal tumor, etc.) or a rare pathological type (nephroblastoma, Ewing sarcoma, etc.) were excluded. Finally, 226 patients were included in this study. The operations were completed by ten senior and experienced urologists in our center. Patients who underwent surgery after May 2019 were set as a validation group (n=60) to verify the prediction model (**Figure 1**).

### Scoring Indicator Determination and Risk Stratification

We included seven preoperative indicators related to the surgical management of RCC with VTT, based on a literature review and extensive clinical experience, to establish a T.H.R.O.B.V.S. grading system (**Table 1**). They are (T)est of alkaline phosphatase (ALP), (H)eight of tumor thrombus, maximum diameter of primary (R)enal tumor, and (O)besity, concomitant (B)land thrombus, (V)ascular wall invasion, (S)ide of the primary tumor. Two experienced radiologists evaluated the imaging data separately, and the third highly qualified radiologist judged if the results were inconsistent.

**Table 1:** T.H.R.O.B.V.S. score content.

T.H.R.O.B.V.S. Grading System				
Abbreviation	Items	Score		
		0	1	2
T	Test of ALP	<ULN	–	>ULN
H	Height of the tumor thrombus (Mayo Grade)	0	I/II	III/IV
R	Renal Cancer (Max diameter, cm)	0-7	7-10	>10
O	Obesity (BMI, kg/m^2^)	<24	24-30	>30
B	Bland thrombus	No	–	Yes
V	Vascular wall invasion	No	–	Yes
S	Side	Right	–	Left

Low-risk: 0-2; Middle-risk: 3-5; High-risk: ≥6.

ULN, Upper Limit of the Normal range; BMI, Body mass index.

It involves seven items, including the ALP level, the height of the tumor thrombus, the maximum diameter of primary renal tumor, obesity, the combined bland thrombus, the vascular wall invasion, and the side. Patients were divided into low, middle, and high-risk groups according to the total score.

ALP refers to alkaline phosphatase within one week before the operation, with 45~125 U/L as the reference value. If the score was higher than the upper limit (ULN), 2 points were recorded; otherwise, 0 points were recorded. According to the Mayo classification, the height of the tumor thrombus was evaluated based on preoperative computed tomography (CT) or magnetic resonance (MR) examinations. We scored 0 points for Mayo grade 0 tumor thrombus, 1 point for Mayo grade I/II tumor thrombus, and 2 points for Mayo grade III/IV (5).

For the maximum diameter of the tumor, we referred to the T classification in the 2017 American Joint Committee on Cancer (AJCC^)^ 8th edition renal cancer TNM staging (14). CT or MR measurements of 0~7 cm was counted as 0 points, 7~10 cm was counted as 1 point, and over 10 cm was counted as 2 points. Obesity mainly depends on the body mass index (BMI). Patients who were lower than 24 kg/m^2^ were given 0 points, 24~30 kg/m^2^ were given 1 point, and more than 30 kg/m^2^ were given 2 points.

Distinguishing bland thrombi from tumor thrombi is critical in evaluating the “B” score. We used enhanced CT or MR to determine whether concomitant bland thrombi were present. The criteria were proposed in a previous study and showed satisfactory sensitivity and specificity (9). There were also studies introducing the imaging features of tumor vascular wall invasion (15, 16). For “bland thrombus” and “vascular wall invasion”, we assigned 2 points for positive cases and 0 points for negative cases. “S” represents the side of the primary tumor, with 0 points on the right side and 2 points on the left side. Finally, the seven subscores were added up to obtain the final T.H.R.O.B.V.S. score.

**Figure 2:**
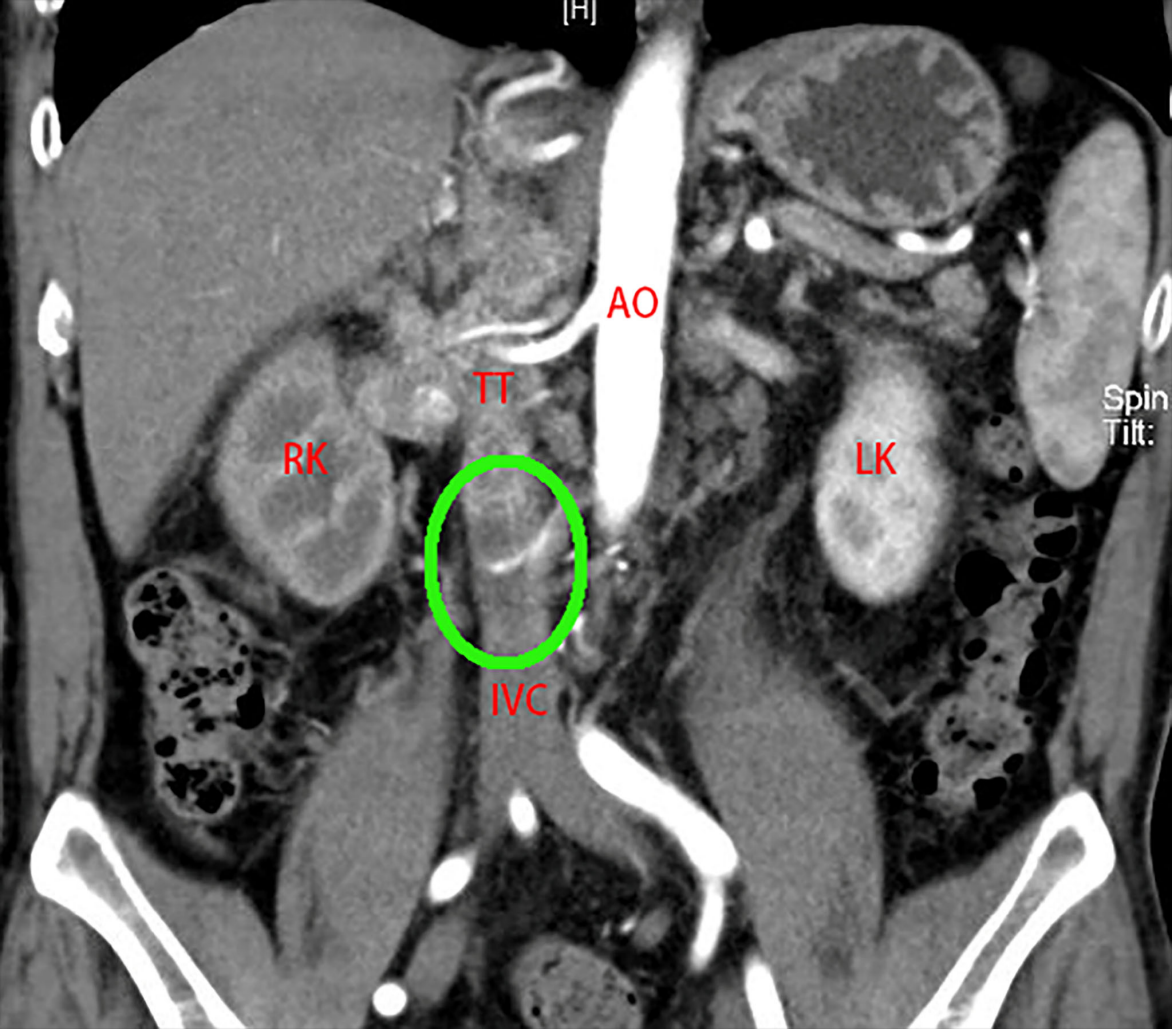
A 53-year-old man was admitted to the hospital with “painless gross hematuria for 3 months”. CTU examination revealed a tumor of the right kidney (maximum diameter 3 cm). The right RV, IVC (below the second hepatic portal) and part of the left RV were widened with uneven enhancement, TT considered. The boundary between TT and the venous wall was unclear, and vascular wall invasion was considered. Bland thrombus formed at the distal end of the IVC (green circle). T.H.R.O.B.V.S. score: 0 + 2+0+1+2+2+0 = 7 points, belonging to the high-risk group. AO, aorta; RK, right kidney; LK, left kidney; IVC, inferior vena cava; TT, tumor thrombus.

According to the total score, we defined 0~2 as a low-risk group, 3~5 as a middle-risk group, and higher than 5 as a high-risk group (**Table 1**). For example, Patient No. 48 (53-year-old male, BMI 26.03 kg/m^2^) was found to have a tumor in the right kidney (maximum diameter 3 cm) combined with a Mayo II VTT (**Figure 2**). The tumor thrombus had invaded the IVC wall, and a bland thrombus was formed at the distal end. The patients preoperative ALP was 103 U/L. Therefore, the T.H.R.O.B.V.S. score was 0+2+0+1+2+2+0 = 7 points, and the patients was classified into the high-risk group.

### Post-Operative Complication and Follow-Up

Postoperative complications were classified by the Clavein-Dindo grading system, in which ≥grade 2 was defined as notable complications where medical interventions were needed. The patients were followed up for the first time one month after the operation, every three months in the first two years, every six months after two years, and once a year after five years. Follow-up was carried out by outpatient revisit and phone call, and the last date was October 2020.

### Statistical Analysis

We used the Kolmogorov Smirnov test to determine the normality of the data. The normally distributed continuous variables were shown as “mean ± standard deviation”, and compared with the analysis of variance (ANOVA). For skewed distributed data, “(median, interquartile range)” and Kruskal–Wallis H (KW-H) tests were chosen. Categorical variables were expressed by frequency (constituent ratio), and the difference was analyzed with the Pearson chi-square test. All data statistics and chart design were completed with SPSS 18.0 (IBM Inc., Chicago, IL, USA) and GraphPad Prism 8 (GraphPad Software Inc., San Diego, CA, USA). P<0.05 was considered a significant difference.

## Results

In the study cohort of 226 cases, 166 cases were set as a trial group, and 60 cases were in the validation group. The clinicopathological and imaging data are shown in **Table 2**. The T.H.R.O.B.V.S. scoring system was established with the trial group. Thirty-two patients (19.28%) were included in the low-risk group (total score ≤ 2 points), 89 patients (53.61%) were included in the middle-risk group (total score 3~5 points), and 45 patients (27.11%) were included in the high-risk group (total score ≥6 points). Surgical approaches (complete laparoscopic surgery, open or conversion to open surgery), operation time, intraoperative blood loss volume, and blood and plasma transfusion were compared among the three groups as indicators of surgical complexity (**Table 3**).

**Table 2:** Clinical and pathological data of patients in each group.

Characteristic	Value	
	Trial group (N = 166)	Validation group (N = 60)
Sex, n (%)		
Male	126 (75.90%)	42 (70.00%)
Female	40 (24.10%)	18 (30.00%)
Age, y	59.22 ± 10.29	61.62 ± 11.96
BMI, kg/m^2^	23.63 ± 3.71	24.11 ± 3.85
ASA score, n (%)		
1	11 (6.63%)	3 (5.00%)
2	130 (78.31%)	50 (83.33%)
3	25 (15.06%)	7 (11.67%)
Surgical approach, n (%)		
Laparoscopic approach	81 (48.80%)	32 (53.33%)
Open approach	85 (51.20%)	28 (46.67%)
Tumor diameter, cm	8.22 ± 3.51	8.37 ± 2.53
Tumor height (Mayo grade), n (%)		
Mayo 0	42 (25.30%)	22 (36.67%)
Mayo I	40 (24.10%)	8 (13.33%)
Mayo II	49 (29.52%)	23 (38.33%)
Mayo III	19 (11.45%)	4 (6.67%)
Mayo IV	16 (9.64%)	3 (5.00%)
Alkaline phosphatase, U/L	96.78 ± 48.33	106.92 ± 105.59
cN stage, n (%)		
cN0	67 (40.36%)	20 (33.33%)
cN1	99 (59.64%)	40 (66.67%)
cM stage, n (%)		
cM0	125 (75.30%)	45 (75.00%)
cM1	41 (24.70%)	15 (25.00%)
Combined bland thrombus, n (%)		
Yes	20 (12.05%)	14 (23.33%)
vNo	146 (87.95%)	46 (76.67%)
Vascular invasion, n (%)		
Yes	64 (38.55%)	27 (45.00%)
vNo	102 (61.45%)	33 (55.00%)
Side, n (%)		
Left	57 (34.34%)	28 (46.67%)
Right	109 (65.66%)	32 (53.33%)
Operation time, min	320, 182	302, 154
Blood loss volume, ml	600, 1800	600, 900
Blood transfusion, ml	0, 1600	400, 800
Plasma transfusion, ml	0, 150	0, 400
Hospitalization duration, days	9, 7	8, 5
Complication (Clavein-Dindo grading), n (%)		
I	5 (3.01%)	3 (5.00%)
≥II	42 (25.30%)	8 (13.33%)
Nuclear grade, n (%)		
I	3 (1.81%)	1 (1.67%)
II	58 (34.94%)	20 (33.33%)
III	67 (40.36%)	28 (46.67%)
IV	38 (22.89%)	11 (18.33%)

Normally distributed data are presented as the mean ± SD; data in a skewed distribution pattern are shown as (median, IQR). BMI, body mass index; ASA, American Society of Anesthesiologists; SD, standard deviation; IQR, interquartile range.

**Table 3:** Comparison of parameters related to operation complexity and postoperative management among risk groups.

Item	Group			F	χ^2^	H	P value
	Low-risk (n = 32)	Middle-risk (n = 89)	High-risk (n = 45)				
Age	59.28 ± 9.90	59.33 ± 9.29	58.96 ± 12.50	0.021			0.979
Sex					0.415		0.812
Male	23 (71.88%)	69 (77.53%)	34 (75.56%)				
Female	9 (28.13%)	20 (22.47%)	11 (24.44%)				
Operating approach					28.721		<0.001*
Laparoscopic	26 (81.25%)	46 (51.69%)	9 (20.00%)				
Open	6 (18.75%)	43 (48.31%)	36 (80.00%)				
Operation time (min)	273, 140.75	286, 142.00	421, 142.50			50.629	<0.001*
Intraoperative blood loss volume (ml)	225.00, 875.00	500.00, 1000.00	2300.00, 3100.00			42.697	<0.001*
RBC transfusion (ml)	0.00, 400.00	0.00, 800.00	1600.00, 2400.00			37.596	<0.001*
Plasma transfusion (ml)	0.00, 0.00	0.00, 0.00	400.00, 800.00			32.106	<0.001*
Post-operative Complication					22.672		<0.001
No	25 (78.13%)	66 (74.16%)	16 (35.56%)				
Yes	7 (21.88%)	23 (25.84%)	29 (64.44%)				
Notable complication					26.577		<0.001*
No	27 (84.38%)	69 (77.53%)	17 (37.78%)				
Yes	5 (15.63%)	20 (22.47%)	28 (62.22%)				
Post-operative hospital stay (days)	8, 4.00	8, 5.00	13, 5.50			27.840	<0.001*
Nuclear grade					7.164		0.306
I	0 (0.00%)	3 (3.37%)	0 (0.00%)				
II	13 (40.63%)	33 (37.08%)	12 (26.67%)				
III	14 (43.75%)	35 (39.33%)	18 (40.00%)				
IV	5 (15.63%)	18 (20.22%)	15 (33.33%)				

Normally distributed data are presented as the mean ± SD; data of other distribution patterns are shown as (median, IQR). RBC, red blood cell. F, statistic in one-way analysis of variance (ANOVA). χ^2^, statistic in Chi-square test. H, statistic in Kruskal−Wallis H test. *, significant difference detected both in trial group and validation group; SD, standard deviation; IQR, interquartile range.

**Figure 3:**
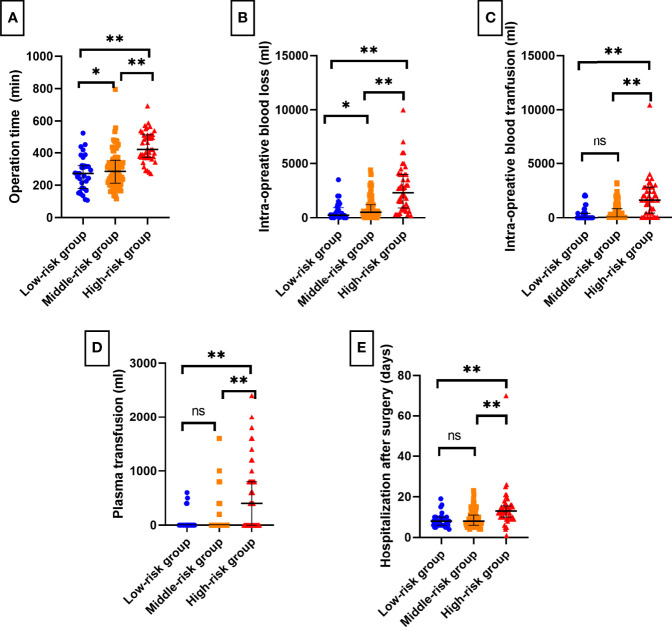
Bar charts of surgical complexity indicators and short-term postoperative recovery among T.H.R.O.B.V.S. risk stratifications. The grading system had a good effect on predicting **(A)** operation time, **(B)** intraoperative blood loss, **(C)** blood transfusion, **(D)** plasma transfusion, and **(E)** postoperative hospital stays. ns, no significant difference; *P < 0.05; **P < 0.01.

In the low-, middle-, and high-risk groups, 81.25%, 51.69%, and 20.00% of patients underwent laparoscopic surgery, respectively, and the proportion of patients who received open surgery increased with the increase in the T.H.R.O.B.V.S. score, with a significant difference (P < 0.001). The median operation times of the three groups were 273 min, 286 min, and 421 min, respectively. As the risk group increased, the operation time was significantly prolonged (P<0.001), the median intraoperative blood loss volume was significantly increased (225 ml vs. 500 ml vs. 2300 ml, P<0.001), and the intraoperative blood transfusion volume in the high-risk group was significantly higher than that of the other two groups (0 ml vs. 0 ml vs. 1600 ml, P<0.001). There was a similar significant difference in the amount of plasma transfusion (0 ml vs. 0 ml vs. 400 ml, P<0.001). All of these differences or trends were verified in the validation group. There was also a significant difference in the length of hospital stay after the operation (P<0.001, **Table 3** and **Figure 3E**).

The postoperative complications in each group are shown in **Table 4**. Seven cases (21.88%) had postoperative complications in the low-risk group, including 5 cases (15.63%) with notable complications. They were apparent lymphatic leakage, lower extremity thrombosis, pulmonary infection, anemia, and incomplete intestinal obstruction. Most cases relieved after anti-infection, blood transfusion, and gastrointestinal decompression. There was also a case of acute renal failure after the operation, which was cured by hemodialysis. Twenty-three complications (25.84%) were reported in the middle-risk group, twenty cases (22.47%) of which were notable complications, with a higher prevalence of lymphatic leakage, thrombosis, infection, and renal failure. Twenty-nine cases (64.44%) of complications happened in the high-risk group, and 28 cases (62.22%) with notable complications, where two of them died of multiple organ failure. No patient underwent reoperation due to perioperative complications. There were significant differences in the incidence of postoperative complications among the three groups (P<0.001), as well as notable complications (P<0.001). However, no statistically significant difference was detected in the validation group (P=0.092 vs. P=0.197). The median hospital stays in the low-, middle-, and high-risk groups were 8 days, 8 days, and 13 days, respectively, and the difference was statistically significant (P<0.001). However, there was no significant difference between the three groups regarding postoperative pathology or Fuhrmann grading.

**Table 4:** Postoperative complications in different T.H.R.O.B.V.S. groups.

	Low-risk n = 32	Middle-risk n = 89	High-risk n = 45	Total
Skin rash	0	1	0	1
Lymphatic fistula	1	3	4	8
Venous thrombosis of lower extremity	1	3	4	8
Pulmonary infection	1	4	2	7
Anemia (Blood transfusion needed)	2	5	7	14
Renal failure	1	3	4	8
Cerebral infarction	0	1	0	1
Heart failure	0	0	1	1
Pleural effusion	0	1	1	2
Incision infection	0	1	1	2
Death	0	0	2	2
Others	1	1	2	4
Total	7	23	28	58

## Discussion

RCC is a malignant tumor with a high mortality rate in the urinary system, and the prognosis of patients with VTT is even worse (1, 17). Patients who undergo RN and tumor thrombectomy can obtain significant survival benefits (4, 18). The main steps of this operation include the complete removal of the affected kidney (including perirenal fat and Gerota’s fascia) and removal of the VTT. The increased difficulty of any step leads to the greater overall complexity. For tumor size (R score), large primary tumors tend to adhere to surrounding tissues more tightly. Sharp separation is necessary while freeing the kidney, and more tissues need to be dissociated to obtain enough operating space. It results in a long operation time, more blood loss, and a higher probability of postoperative complications. Similarly, the properties of the VTT (H score) also play a role in surgical complexity (10).

Current research shows that the operation time of obese patients (O score) is significantly prolonged due to the thickened fat of the abdominal wall and retroperitoneal cavity, making the surgical field not well exposed (19). The postoperative drainage tube retention time, drainage volume, and postoperative hospitalization days are prolonged (20). Moreover, obese patients may have a variety of underlying diseases, such as hypertension, diabetes, and heart disease. The incidence of postoperative wound infection and liquefaction increased, complicating perioperative management.

Previous studies have also reported the adverse effects of the combined bland thrombus (B score) and vascular wall invasion (V score) on the operation of renal cancer thrombus (16). The reasons may be as follows: (1) The bland thrombus is more often observed in patients with high-level tumor thrombus than that in lower-level tumor thrombus patients (95.2% vs. 39.2%) (16, 21); (2) Most of the blood vessels in patients with bland thrombus were blocked entirely. The Foley catheter method and the “Milking” method were usually ineffective; (3) The occlusion of blood vessels causes the collateral circulation, increases the tumor blood supply, and increases the amount of intraoperative blood loss. For cases of vascular wall invasion, aggressive procedures, including venous wall resection and anastomosis, segmental IVC resection, and even artificial blood vessel replacement, are required, which dramatically increases the complexity of the operation.

Existing models for surgical complexity prediction primarily consider anatomical-related factors, such as the R.E.N.A.L. scoring system (22). However, RCC with VTT is classified as T3a or higher stage. More attention should be given to laboratory tests due to their high sensitivity to tumor microenvironment alterations, leading to the detection of abnormal clues earlier than imaging studies. Liu et al. retrospectively analyzed 123 patients and found that metastasis and elevated ALP were independent risk factors of poor prognosis for patients with RCC and VTT (16). Another study carried out by Borregales et al. showed similar results (23), but the underlying mechanism needs to be further elucidated, which may be related to micro bone metastasis (14, 24). Moreover, elevated ALP and AST mean liver damage and decreased coagulation function, creating a high potential of perioperative hemorrhage, and more careful hemostasis is required during the operation. Therefore, the operation time is prolonged, and the perioperative treatment is complicated (24).

As for the side (S) score, the operation of the left renal tumor is more complex because the anatomy of the gonadal vein is different between two sides. For right RCC operation, the blood flow of the left renal artery can still return to the venous system through the gonadal vein. However, the blood of the right renal artery cannot return to the vein during the left RCC operation due to the lack of bypass vessels. The warm ischemia time is prolonged, which requires advanced surgical techniques. In addition, the left renal vein is long, and the superior mesenteric artery is relatively fixed, so it is challenging to complete all operations in one posture (25).

The results indicated that the T.H.R.O.B.V.S. scoring system had an excellent stratification effect on the surgical complexity of RCC with VTT (**Figure 3**), which was verified in the independent validation group. Although there was no significant difference in the incidence of postoperative complications in the validation group, there was still a similar trend (P = 0.092), which may be related to the small number of cases in this group. As the risk level increased, the proportion of cases with notable complications increased. It indicates that patients in the high-risk group may have more severe complications, and adequate preparations must be made before surgery. These patients are recommended to be transferred to the ICU ward for more rigorous monitoring after surgery.

In the pairwise comparison of risk groups, most indicators were significantly different, except blood transfusion volume and plasma transfusion volume between the low-risk group and middle-risk group (**Figures 3C, D**). However, there were significant differences between them and the high-risk group. The reasons may be as follows: (1) the score of the high-risk group was composed of at least two items of 2 scores and an item of 1 score (2+2+1+others) or three items of 2 scores (2 + 2 + 2 +others). The combination of multiple risk factors made the operation more complicated. Segmental resection of IVC and resection of metastatic foci may be considered, leading to higher intraoperative blood loss (**Figure 3B**). Moreover, these patients were more likely to have anemia after the operation. Therefore, surgeons may choose intraoperative blood or plasma transfusion to reduce postoperative complications. (2) The results were diluted due to the large range of patients included. Subsequent studies may analyze the specific differences between groups and optimize the size of risk groups.

The study results showed significant differences in postoperative complications among risk groups. RN with thrombectomy is one of the most challenging procedures in urology. Ebbing et al. reported that the incidence of early postoperative complications was as high as 58.6%, and the mortality rate was 3%-16% (26). As mentioned before, the T.H.R.O.B.V.S score simultaneously involves the parameters related to nephrectomy and thrombectomy, and it also has a convincing effect on predicting postoperative complications.

The hemorrhage was the most common complication in this study, especially in patients with high Mayo grade (high H score) and vascular wall invasion (high V score). If an uncontrollable intraoperative hemorrhage occurs, the surgeon should try to clamp the aorta above the celiac trunk or asks a cardiac surgeon to initiate CPB (17). However, the CPB may cause coagulation dysfunction, prolong operation time, and increase intraoperative bleeding, thus greatly increasing the difficulty of operation. In addition, elevated pre-operative ALP and AST levels (high T-score), which may reflect hepatic dysfunction owing to suprahepatic IVC obstruction, were associated with a greater risk of major complications and worse overall survival (24). Large quantities of fresh frozen plasma, platelets, and red blood cells should be considered for transfusion to correct anemia.

Postoperative venous thrombosis of lower limbs is also common. The possible reasons are as follows: (1) postoperative long-time bed rest and reduced limb activity caused by severe surgical trauma; (2) Massive intraoperative bleeding and insufficient postoperative rehydration caused thrombosis. Therefore, patients should be encouraged to exercise their lower limbs or go to the ground early, and wearing elastic socks is also a good choice. The application of low molecular weight heparin can also prevent the formation or progression of lower extremity thrombosis.

Renal insufficiency or acute renal failure are also noteworthy, which may be related as follows: (1) renal ischemia caused by contralateral renal vein occlusion during operation, or (2) prerenal renal failure caused by massive hemorrhage and insufficient circulating capacity during operation. A total of 8 cases of renal failure complicated with hyperkalemia were reported in the study. Two patients did not improve after rehydration and diuretic treatment in the high-risk group. Therefore, hemodialysis was used. The blood creatinine decreased to the preoperative level one month later. In addition, another complication, lymphatic leakage, is mainly caused by the damage of lymphatic vessels during lymph node dissection. Most patients can be relieved after fasting, but the time of removing the drainage tube should be delayed, which increases the length of hospital stay.

The highlight of the T.H.R.O.B.V.S. scoring system is that it quantifies the preoperative condition of patients with RCC combined with VTT from multiple aspects, endows patients with risk stratification, and provides clinicians with a set of convenient guidance for surgical decisions and perioperative preparation. This study showed that the patients in the low-risk group had relatively fewer surgical procedures, lower technical requirements for surgeons, less intraoperative bleeding and blood transfusion, and satisfactory short-term postoperative recovery. However, with the increase in risk stratification, more patients choose traditional open surgery. Advanced techniques have been adopted according to the tumor and tumor thrombus characteristics, with higher technical requirements for surgeons. For example, a higher Mayo grade of tumor thrombus means a broader range of blood flow control and even requires multidisciplinary cooperation to help overturn the liver, cut open the diaphragm or establish cardiopulmonary bypass; tumor thrombus with tight vascular adhesion often needs sharp separation, segmental vein resection or artificial vascular implantation (4, 17, 27). The above processes increase the complexity of the operation, prolong the operation time, and increase blood loss.

Therefore, it is suggested that more detailed preparation should be made before the operation for patients in the middle- and high-risk groups. (1) Multidisciplinary consultation should be organized to discuss the possible surgical plan, determine the scope of vascular occlusion, apply special techniques, and select the appropriate surgical approach. (2) The operation should be performed or guided by experienced surgeons. (3) Sufficient blood products should be prepared for patients in the high-risk group before the operation. (4) Full communication with patients before the operation is necessary. Patients should be informed of the complexity and greater risk of this operation, higher possibility of postoperative complications, and more extended hospital stays. (5) The ICU ward should be reserved for close monitoring after the operation. Previous research proposed the PKUTHLP score to assist in the surgical approach choice for patients with RCC combined with VTT and PKUTH scores to predict intraoperative blood loss (28, 29). Surgeons would undertake more thoughtful perioperative preparations with the help of the T.H.R.O.B.V.S. score and these tools.

Although the T.H.R.O.B.V.S. score imaging evaluation can be mostly completed by CT alone, this does not mean that other imaging examinations can be omitted. Jae et al. pointed out that a complete and detailed imaging evaluation is necessary to formulate surgical strategies (30), which is why senior urologists are often required to participate (13, 31). We recommend the following before formulating a comprehensive treatment plan for patients: (1) Detailed consultation and physical examination; (2) Urinary ultrasound: Patients with Mayo grade IV tumor thrombus should undergo preoperative echocardiography and intraoperative transesophageal ultrasound; (3) Plain and enhanced CT urography; (4) Plain and enhanced MRI of IVC within one week before operation to determine the level of tumor thrombus; (5) IVC angiography can be considered in patients with severe IVC occlusion and formation of collateral circulation to determine the extent of tumor thrombus and collateral circulation, to reduce intraoperative blood loss. If the tumor thrombus seriously invades the vascular wall and the formation of collateral circulation is good, segmental resection of IVC can be considered to eradicate the tumor completely, but the complexity of operation increases accordingly (11, 13).

The results show that the T.H.R.O.B.V.S. scoring system has promising predictive effects on surgical complexity and is easy to operate. However, the results should be interpreted in light of limitations. (1) The study was retrospectively designed and based on single-center data. Multicenter studies are necessary for the future to further evaluate the predictive power of the scoring system and enhance the veracity of the conclusion due to the rare nature of RCC and VTT. (2) The study is based on clinical experience. They have not undergone rigorous univariate and multivariate analysis, Cox analysis, nomogram, and other statistical steps. (3) Surgical complexity indicators, including operation time and intraoperative blood loss, may be affected by other factors, such as the surgeon’s experience and different hemostatic equipment and methods. The operations were performed by 10 senior surgeons, who have higher surgical skills and more experience, so the model may underestimate the complexity of the operation for lower-level surgeons. Therefore, although the T.H.R.O.B.V.S. scoring system sacrifices part of the accuracy, it is convenient and feasible for surgeons to make reproducible clinical decisions, and it has excellent potential in the guidance of perioperative preparation and doctor–patient communication.

## Conclusion

T.H.R.O.B.V.S. scoring system is a multiparameter quantifiable tool for predicting the surgical complexity of patients with RCC combined with VTT. It evaluates the patient’s condition comprehensively and conveniently and is significant for surgical decisions, perioperative preparation, and doctor–patient communication.

## Data Availability Statement

The original contributions presented in the study are included in the article. Further inquiries can be directed to the corresponding authors.

## Ethics Statement

The studies involving human participants were reviewed and approved by Ethics Committee of Peking University Third Hospital. Written informed consent for participation was not required for this study in accordance with the national legislation and the institutional requirements.

## Author Contributions

KW: Data curation, formal analysis, conceptualization, manuscript drafting and proofing. ZL: Data curation, formal analysis, conceptualization, manuscript drafting and proofing. PH: Data curation, methodology guidance, manuscript editing. YCQ: Data curation, statistical guidance, manuscript drafting. XZ: Data curation, quality control, methodology and statistical guidance. HXZ: Formal analysis, conceptualization, manuscript proofing. CL: Data curation, methodology guidance, proofed and revised article. LYG: Designed the study, data curation, proofed and revised article, analyzed data. LLM: Designed the study, supervision, data curation, proofed and revised article. All authors contributed to the article and approved the submitted version.

## Funding

This work was supported by the grants from the National Natural Science Foundation of China (Nos. 81972381 and 82173385).

## Conflict of Interest

The authors declare that the research was conducted in the absence of any commercial or financial relationships that could be construed as a potential conflict of interest.

## Publisher’s Note

All claims expressed in this article are solely those of the authors and do not necessarily represent those of their affiliated organizations, or those of the publisher, the editors and the reviewers. Any product that may be evaluated in this article, or claim that may be made by its manufacturer, is not guaranteed or endorsed by the publisher.

## Abbreviation

RCC, Renal cell carcinoma; VTT, Venous tumor thrombus; IVC, Inferior vena cava; RN, Radical nephrectomy; ALP, Alkaline phosphatase; ULN, Upper Limit of the Normal range; CT, Computed tomography; MR, Magnetic resonance; AJCC, American Joint Committee on Cancer; BMI, Body mass index; ANOVA, Analysis of variance; KW-H, Kruskal Wallis H test; CSS, Cancer-specific survival; ICU, Intensive care unit.
